# Non-invasive imaging techniques in assessing non-alcoholic fatty liver disease: a current status of available methods

**Published:** 2017

**Authors:** AM Lăpădat, IR Jianu, BS Ungureanu, LM Florescu, DI Gheonea, S Sovaila, IA Gheonea

**Affiliations:** * Radiology and Medical Imaging, University of Medicine and Pharmacy, Craiova, Romania; ** Research Center of Gastroenterology and Hepatology, University of Medicine and Pharmacy, Craiova, Romania; *** Centre Hospitalier de Sedan, France and Internist.ro Clinic, Brasov, Romania

**Keywords:** non-alcoholic fatty liver disease, non-invasive imaging techniques, hepatosteatosis

## Abstract

Non-alcoholic fatty liver disease (NAFLD) is an ailment affecting and increasing a number of people worldwide diagnosed via non-invasive imaging techniques, at a time when a minimum harm caused by medical procedures is rightfully emphasized, more sought after, than ever before. Liver steatosis should not be taken lightly even if its evolution is largely benign as it has the potential to develop into non-alcoholic steatohepatitis (NASH) or even more concerning, hepatic cirrhosis, and hepatocellular carcinoma (HCC).

Traditionally, liver biopsy has been the standard for diagnosing this particular liver disease, but nowadays, a consistent number of imagistic methods are available for diagnosing hepatosteatosis and choosing the one appropriate to the clinical context is the key. Although different in sensitivity and specificity when it comes to determining the hepatic fat fraction (FF), these imaging techniques possessing a diverse availability, operating difficulty, cost, and reproducibility are invaluable to any modern physician. Ultrasonography (US), computed tomography (CT), magnetic resonance imaging (MRI), elastography, and spectroscopy will be discussed in order to lay out the advantages and disadvantages of their diagnostic potential and application.

Although imagistics has given physicians a valuable insight into the means of managing NAFLD, the current methods are far from perfect, but given the time, they will surely be improved and the use of liver biopsy will be completely removed.

## Introduction

Non-alcoholic fatty liver disease (NAFLD) represents a hepatic lipid accumulation at an intra-cellular level, over time [**1**]. With more than 20% to 30% of the world population being affected, NAFLD oversees a wide range of hepatic disorders and is frequently associated with diabetes mellitus, dyslipidemia, obesity and hypertension, joining up to form a metabolic syndrome [**2**-**4**]. The disease may present as an incidental finding in patients with a persistent abnormal level of hepatic enzymes or during an abdominal ultrasound. Even though NAFLD may have a benign evolution, in 5-6% of the cases, it might lead to non-alcoholic steatohepatitis (NASH) [**5**] and to the development of even more serious conditions [**4**,**6**,**7**], such as cirrhosis and hepatocellular carcinoma (HCC).

Confirming the presence of NAFLD and controlling the disease’s evolution is conditioned by the assessment of the liver’s fat content. A quick diagnosis is imperative, as steatosis has only one documented management method which consists of lifestyle changes focusing on nutrition and exercise [**5**]. Liver biopsy is known to be the trademark for the diagnosis and classification standards of NAFLD. However, this procedure should be used only when there is a lack of more beneficial alternatives, due to its invasiveness and potential for error upon the retrieval of the tissue samples. Secondly, hepatosteatosis is a heterogenic disease, and may be difficult to pathologically lead to a diagnosis, because the biopsy samples only reflect a small portion of the hepatic cellular architecture [**8**-**10**]. Even so, the pathological examinations of cross-liver sections and the semi-quantitative estimation of the percentage of hepatic parenchyma cells containing fat droplets is widely used as a conventional mean of determining the degree of hepatosteatosis [**5**,**10**]. 

Currently, there is a justified high emphasis on causing as little discomfort as possible, with a global focus on less invasive techniques, with minimal risks to the patient’s wellbeing. Striving to overcome the risks of invasive procedures, imaging techniques are on the spotlight in diagnosing and assessing liver steatosis. Several methods have received an extensive attention and have proven to be worthy of a future evaluation such as ultrasonography (US), elastography, computed tomography (CT), magnetic resonance imaging (MRI), magnetic resonance spectroscopy (MRS) and chemical shift imaging (CSI). With such a large demand for quality screening, diagnosis and treatment assessment for patients suffering from hepatic steatosis, imaging methods seem to be the future direction of managing NAFLD. Therefore, we tried to review the current standings of the NALFD non-invasive diagnosis and management from the available methods to more advanced techniques (**Table 1**). 

**Table 1:** Current available methods and availability for liver steatosis assessment

Technique	Availability	Patient risk	Procedure duration	Operating difficulty	Procedure cost	Accuracy in determining hepatic lipid content	Reproducibility
*US*	High	None	Low	Low	Low	Low to mild hepatosteatosis	Fair
*CT*	High	Possible radiation hazard	Fair	Low	Fair	Low to mild hepatosteatosis	Fair
*MRI*	Fair	None	High	Fair	High	High	High
*MRS*	Low	None	High	High	High	High	High
*US = Ultrasonography; CT = Computed tomography; MRI = Magnetic resonance imaging; MRS = Magnetic resonance spectroscopy*							

**Ultrasonography (US)**

**Fig. 1:**
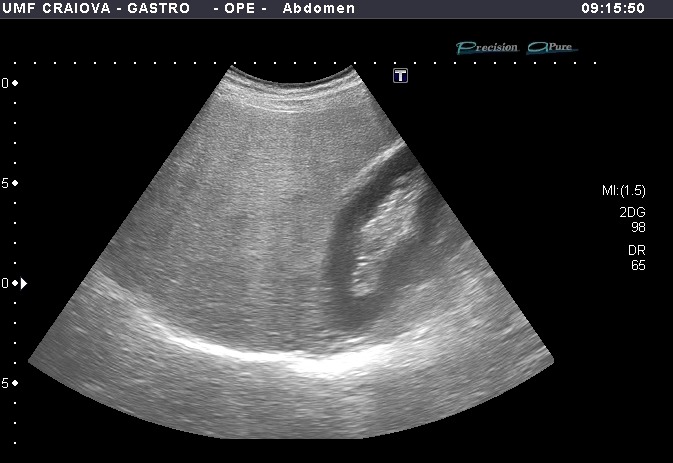
B-mode ultrasound showing hyperechoic liver comparing with the kidney parenchyma and posterior attenuation of the deep liver parenchyma in the context of hepatic steatosis

Usually, US is the first frontier in diagnosing NAFLD in patients with elevated hepatic enzymes. Even though it may not be as precise as liver biopsy, US surely has stronger advantages being a non-invasive technique, with no radiation, available in extremely high numbers across the medical centers across the world and with low costs [**11**-**13**]. Normal hepatic tissue is considered homogeneous in echotexture, similar to the right kidney’s cortex and the spleen’s parenchyma. Through the intracellular build-up of fat droplets, NAFLD on US imaging is identified by a higher echogenicity than the other two reference organs (**Fig. 1**). 

Moreover, portal or hepatic vein vascular blurring and hepatomegaly may accompany hepatic steatosis [**11**,**14**-**16**]. For a proper US exam, a 2-5 MHz convex probe should be used. Usually, there are three levels of hepatosteatosis utilized, ranging from mild to severe [**16**-**20**] and in case of optimal gain settings, they accurately describe the echogenicity features (**Table 2**). Considering the percentage of hepatocytes affected by liver steatosis the US is used for grading (**Table 3**).

**Table 2:** Characteristics and classification of hepatic steatosis

Hepatic echogenicity	Level of hepatosteatosis
Normal	0
Simply increased	1 (mild)
Echogenic walls of portal vein branches obscured by liver echogenicity	2 (moderate)
Diaphragmatic outline obscured by liver echogenicity	3 (severe)

**Table 3:** Grading of hepatic steatosis

grade 0 (normal) = up to 10% of the cells affected;
grade 1 (mild) = 10–33% of the cells affected;
grade 2 (moderate) = 34–66% of the cells affected;
grade 3 (severe) = ≥ 67% of the cells affected;

However, US has several limitations, especially when trying to detect mild levels of fatty liver disease or when trying to distinguish hepatic fibrosis from steatosis [**16**-**21**]. Several studies have demonstrated a 60% sensitivity and 80% specificity for detecting NAFLD [**13**,**19**,**22**-**26**]. Even though US is relatively easy to perform and interpret, some limitations may be encountered: a quantitative assessment is not performed, when lower than 20% steatosis may not be detected [**11**,**27**,**28**], high inaccuracies are related to obese patients and the fact that it is operator dependent may interfere with the results. Therefore, additional research is required to raise US to clinical and technical peaks. This is the moment transient elastography and acoustic radiation force impulse elastography come into play, as they can be integrated into the standard USG system adding to the utility of US.

**Sonoelastography**

**Fig. 2:**
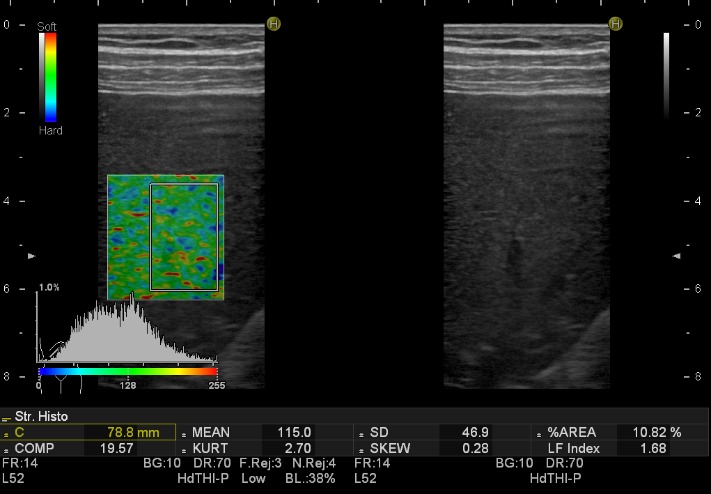
Quantitative (mean hue histogram = 115) and qualitative (soft appearance of the liver parenchyma) real-time elastography revealing hepatic steatosis

Transient elastography (FibroScan, EchoSens, Paris, France) and acoustic radiation force impulse (ARFI) elastography are important tools used to determine the extent of the liver stiffness. Elastography is one of the few imaging methods capable of determining and characterizing liver steatosis and fibrosis [**29**,**30**]. FibroScan translates the degree of fat infiltration of the hepatic parenchyma into the liver stiffness. The higher the tissue rigidity, the faster the sheer wave is propagated [**31**-**33**]. CAP (Controlled Attenuation Parameter) is used to quantify liver steatosis. This technique is coupled with the FibroScan and measures ultrasound wave attenuation, which change depending mainly on the viscosity of the medium through which the waves travel. On the other hand, ARFI is used to examine the elasticity of a certain anatomical area during real-time B-mode imaging, with the help of a region-of-interest cursor. It is similar to FibroScan [**34**]. Real-time elastography (RTE) represents the other imaging technique, and provides real-time measurements of liver stiffness [**35**]. It uses a B-mode machine, incorporating the elastography software into the conventional ultrasound scanner. The relative elasticity of the tissue is calculated and displayed as real-time color images simultaneously with the B-mode images. The disadvantages of this method are the body mass index, the penetration is limited to 3-4 cm and also operator dependency [**36**]. New ultrasound machines have included real time analysis software for the quantitative assessment of the stiffness by using hue histograms. This partially eliminates the human bias (**Fig. 2**).

**Computed tomography (CT)**

A non-operator dependent, fast, but more harmful alternative to US in diagnosing NAFLD is the CT. It involves subjecting the patient to radiation, but radiation exposure can be kept at a minimum by using low-dose protocols. Due to factors [**11**,**14**,**37**] related to scan timing and contrast material enhanced CT is less used in favor of unenhanced CT.

**Fig. 3:**
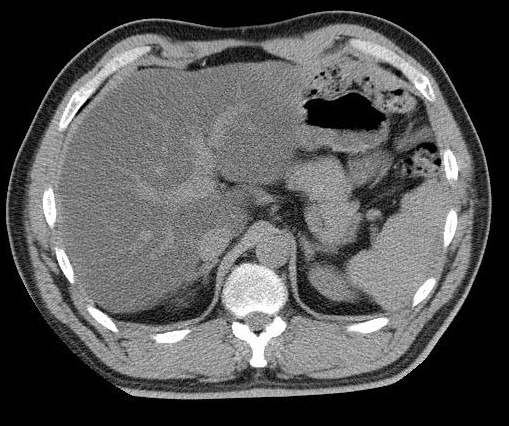
16 slices-unenhanced CT, which depicts lower attenuation values of liver tissue comparing with spleen

NAFLD is determined with a high efficiency by using an unenhanced CT as it evaluates the liver attenuation quantitatively (**Fig. 3**). 

**Fig. 4:**
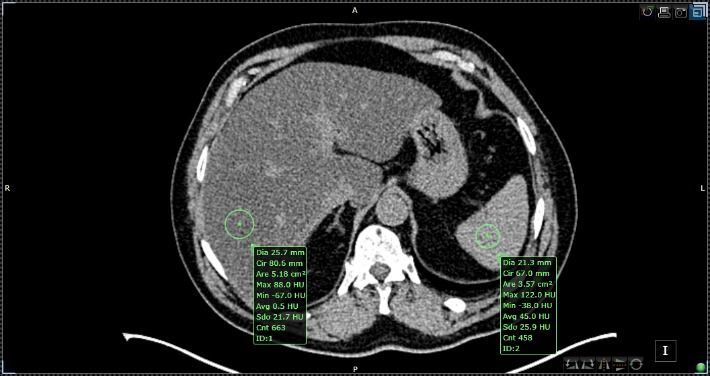
Liver steatosis semi-quantitative assessment with Siemens 16-slices-unenhanced CT with the region of interest (ROI) placement in the liver and spleen parenchyma

Using the aforementioned low-dose protocols, an unenhanced CT (implementing a 10-mm section thickness with a collimation of 128 × 0.625 at 80 kV and 100 mAs with modulated dose) measures liver attenuation by utilizing randomly selected circularly shaped regions of interest (ROIs) ranging from 20 to 40 mm², which may be selected from both lobes (**Fig. 4**). 

The CT evaluation of non-alcoholic fatty liver disease depends on the value of Hounsfield Units (HUs), which represents the attenuation parameters of hepatic parenchyma [**12**,**14**,**15**,**38**]. The normal hepatic tissue has an attenuation value of around 50-65 HU, 8-10 HU higher than a normal spleen [**11**]. Fatty liver assessment may be performed by determining the difference between the attenuation values of liver and spleen, calculating the ratio of absolute measurements in contrast with the liver attenuation index [**38**-**40**]. The lower the attenuation is on the unenhanced CT, the higher the liver lipid content. As a result, the CT method is known to be able to diagnose moderate and severe hepatosteatosis with a specificity of 100% and a sensitivity of 82% [**38**]. Additionally, liver attenuation values of less than 40 HU or a liver-to-spleen attenuation difference > 10 HU confirm the presence of liver steatosis [**13**,**39**,**41**]. Therefore, when the hepatic attenuation is lower than 48 HU, hepatosteatosis can be diagnosed with good accuracy. Studies have shown that liver lipid infiltration of about 30% is translated into a liver attenuation value of 40 HU and that the unenhanced CT diagnosis methods have a more than favorable precision in determining the degree of build-up fat inside the liver. Additionally, this confirmed that the higher liver attenuation values reflect a healthier liver. It has also been suggested that a liver-to-spleen ratio lower than 1 could sometimes predict lipid infiltration [**13**,**39**,**40**]. It can be stated that when hepatic fat build-up is involved, there is a decrease in liver attenuation at low energy levels, which suggests that the tube potential causes an increase in fat attenuation. Regardless of the method used, it is vital to remember that all CT applications imply radiation exposure and just as US, CT has difficulties in picking up mild steatosis. Furthermore, the addition of other pathologies characterized by substance liver accumulation, influence the attenuation of hepatic parenchyma [**42**]. The administration of chemotherapeutics such as methotrexate or amiodarone, other hepatic diseases [**11**,**29**,**43**] including cirrhosis, acute hepatitis or acute toxic hepatic injury also modify the attenuation of the liver’s parenchyma. 

**Magnetic Resonance Imaging**

This particular non-invasive and free of the potential radiation hazard, the MRI, has benefited from numerous technological advancements along the years, but it is still relatively expensive and time consuming. The recent progress in the MRI field has made easier a precise quantitative evaluation of liver steatosis. What makes lipids stand out from the abundant water molecules at a voxel (a value that represents a finite volume in a 3D space) level is a highly specific element, enabled by the main magnetic field [**2**,**44**,**45**], known as the resonance frequency offset.

Today, a large array of techniques come to aid both the physician and the patient (Chemical shift imaging, MR spectroscopy, and MR elastography) and allow the characterization of fatty liver and quantify the fat signal fraction (FSF) or fat fraction (FF), representing liver triglyceride concentration [**45**,**46**]. In the case of modern MR imaging, this biomarker stands for the density of hydrogen protons from fat, normalized from the total hydrogen proton density from all mobile proton species. The value of the FF is modified depending on noise bias, the fat’s spectral complexity, T1 bias, T2* decay and eddy currents [**47**-**51**]. When the fat fraction values are at the upper or lower extremes (close to 0% or 100%), water-fat separation inaccuracies appear, but all this is part of how the eddy currents affect the relative phase of the acquired echoes when using multi-echo acquisitions. This can be avoided through the usage of a magnitude fitting method, in order to mitigate the effects of eddy currents by removing their phase shifts [**50**].

Chemical Shift Imaging (CSI)

The idea of CSI originated from the frequency difference between water and the dominant resonance of methylene within fat and it implies acquisitioning T1-weighted IP (in-phase) and OP (opposed-phase) images. This MRI method is known to have a sensitivity and specificity of 90%, respectively 91% [**51**]. It is based on the fact that lipids and water transverse magnetization vectors develop a phase difference during echo time (TE) and under opposed-phase (OP) conditions results in a shortening of the overall length of the magnetization vector [**52**-**54**]. Measurements for this technique are usually done at values of -220 Hz and 1,5-3 Tesla (T). The level of lipid accumulation is directly related with the amount of signal loss in OP imaging while the signal intensity (SI) for the affected liver is boosted during the IP condition, obtaining a means to determine hepatic fat fraction [**55**,**56**]. Hepatic FF can be determined by calculating the difference between the loss of SI in OP images and SI increase in IP images. This can be simply expressed by using the relationship:

*FF = [(SIip–SIop)/2SIip] x 100*,

where SIip is the liver to spleen signal intensity measured in in-phase images and SIop is the liver to spleen SI in opposed-phase images. Only congruent and acquired within the same breath-hold sets of IP and OP images must be used to obtain a precise FF. Following this method, the hepatic fat content dissemination throughout the liver parenchyma may be estimated while using the obtained FF hepatic maps.

Gradient-echo (GRE) imaging is a type of CSI that involves the patient holding his breath is the most commonly used technique for the determination of the hepatic FSF. Unfortunately, abnormal levels of liver iron, as in hemochromatosis, affects fat fraction when using GRE. It is important to mention that conventional MRI methods only allow the detection of signals from free water and triglycerides. In order to obtain optimal results, rigorous post-processing, proper calibration, and an intricate signal combination from the phased-array coils, spectral modeling alongside T2 correction is necessary. 

The Dixon Technique

**Fig. 5:**
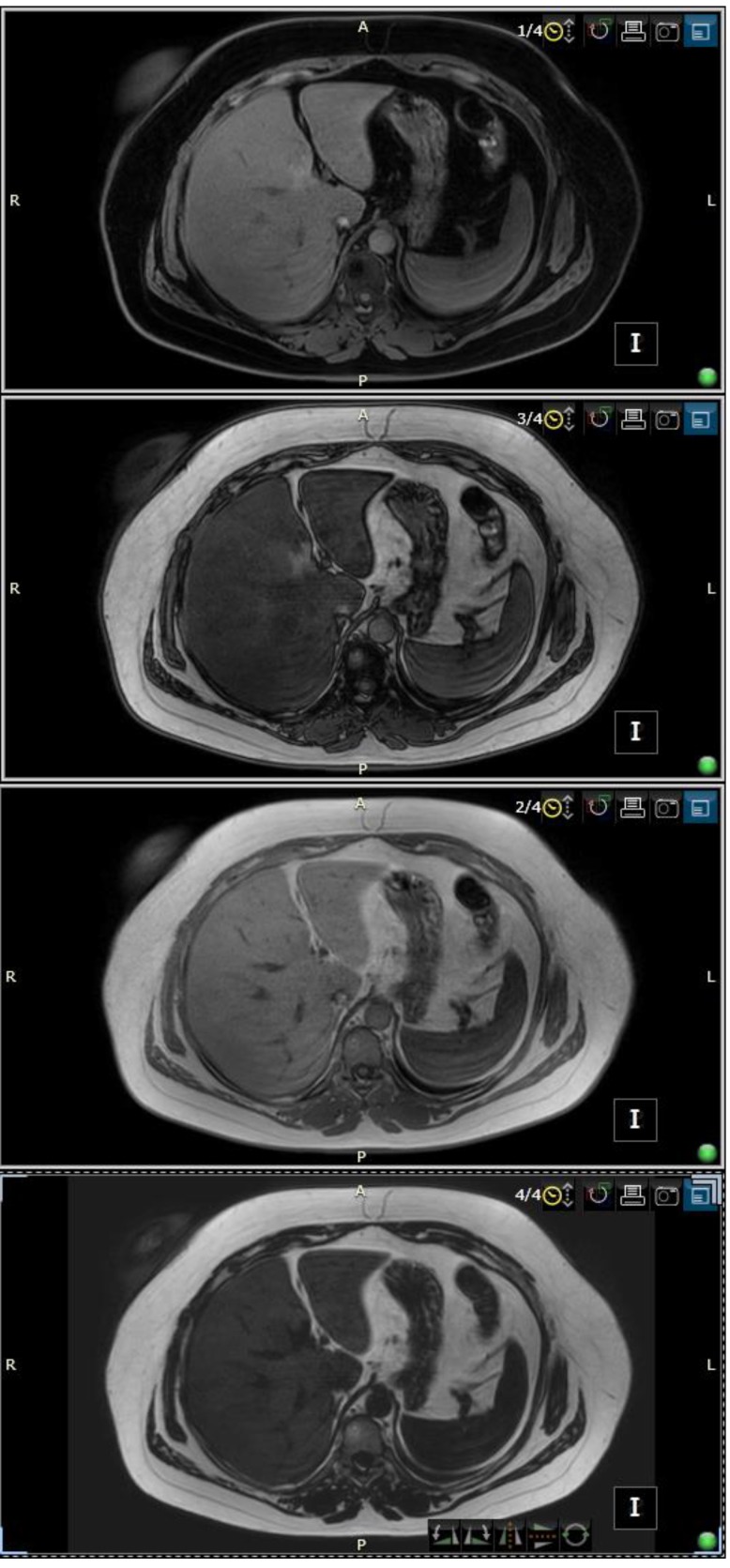
3T MRI, multi-echo DIXON-All breath hold sequences consisting in T1-weighted sequence, in-phase T1 weighted sequence, out-of-phase T1 weighted sequence and fat specific sequence qualitatively assessing liver steatosis

Explained for the first time in 1984 by WT Dixon, this technique initially produced an unacceptable amount of artefacts because of the limited MRI technology at that time [**53**]. Today, this method has been greatly improved and is based on chemical shift, having as a main purpose the uniform fat suppression. Some of the advantages of using this method are better uniformity of the fat signal suppression, fewer artefacts produced, compatible with many other sequence types and weightings, single acquisition images can be provided with and without fat suppression and it is able to quantify the amount of fat (**Fig. 5**).

One of the main characteristics that the Dixon technique has is based on the different rates at which the water and fat molecules are processed. Therefore, the molecules will be found alternating between in-phase and opposed phase. Having said this, the result of running a Dixon technique would be four sequences, after both alternative phases have been acquired [**57**]. While the water only image can only be utilized as a fat suppressed image, the fat only image can then be added to the other sequences of different weightings in order to obtain fat suppression and also for quantification in certain scenarios. However, this method has some limitations when it comes to water-fat interchanging, that are determined by the magnetic field.

*Magnetic Resonance Spectroscopy (MRS)*

In recent years, MR spectroscopy has been considered a reference standard of non-invasiveness when assessing the triglyceride liver content, but not even this method is deprived of complications and is not applicable in every clinical context. MRS is useful for determining the intensity increase of lipid peaks when liver steatosis is present at parts per million (ppm) with values between 1.9 and 2.3, 1.1 and 1.5, and 0.8 and 1.1 [**44**,**58**-**65**]. In the case of in vivo MR spectroscopy, single-T2* correction has been able to produce acceptable results [66], showing an elevated intensity of lipid resonance peak in patients with fatty liver disease. 

**Fig. 6:**
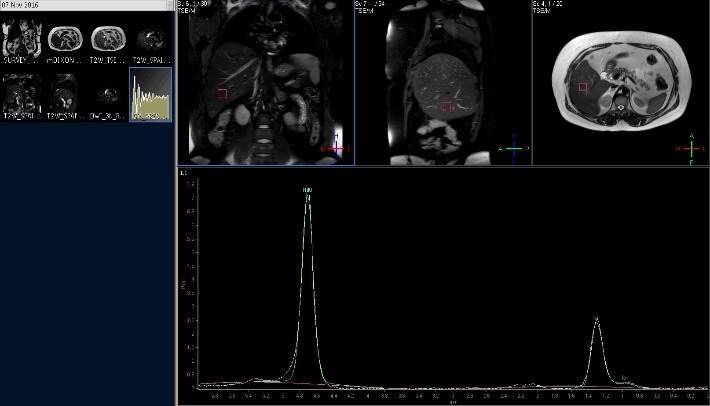
3T single voxel MR spectroscopy quantitatively evaluating liver steatosis with a manually calculated fat-fraction of 42,21% representing moderate steatosis

The collection of spectra for signal fat-fraction estimation requires a proper acquisition technique in order to estimate the fat. Usually, a single voxel is manually placed into the liver parenchyma avoiding liver edges, large vessels, and bile ducts. Through two particular single-voxel MR spectroscopy (SVS) techniques, the FF can be estimated with excellent accuracy: stimulated-echo acquisition (STEAM) and point-resolved spectroscopy (PRESS) [**62**,**64**,**67**,**68**]. The breathing motion must be taken into account because it further complicates conventional MR spectroscopy approaches, while the signal averaging may be lowered or cut out, permitting breath-hold acquisitions. Hepatic water and fat spectra is viewed in high detail and in a direct manner all thanks to high-resolution MR spectroscopy acquisitions. The spectroscopy in the liver is limited to single-voxel acquisition (**Fig. 6**). 

Multivoxel spectroscopy permits the coverage of larger volumes in the liver but the large volume reduces the quality of the shim and spectra and also leads to breathing induced artefacts. 

Every single metabolite benefits from spectral area measurements that can be associated and linked to determine the total fat fraction. The process precision comes from assessing the FF in relation to water. The area of lipid (average peak 1.3) and water (average peak 4.67) need to be recorded to calculate FF. Then, the fat signal is calculated as the fat signal divided by the sum of the water and fat peaks areas with dynamic range results from 0–100%.

However, conventional MR spectroscopy has its disadvantages. For high-resolution readings of small volumes, a process of signal averaging for an optimal signal-to-noise ratio (SNR) and lipid peak separation must be applied to the acquisition. Analyzing the spectra is a less attractive aspect of the MR spectroscopy, as extracting water and lipid quantities requires sophisticated processing software. A spectral-frequency representation is obtained after post-processing and the total ROI lipids are estimated while treating multiple lipid spectra as one element. This approach was proven effective in vivo and in phantoms [**5**,**12**,**13**,**45**]. High-resolution liver MR spectroscopy has proven that lipid spectrum consists of at least six distinct observable triglyceride proton resonances. Thus, the total hepatic lipid fraction is formed by multiple fat spectra that have a role within the MR signal model. 

The empirical evidence offers the values of distinct chemical shifts, leaving the individual’s absolute magnitude differ from sample to sample. Fortunately, calibration is used to obtain relative amplitude values in the case of lipids related to hepatosteatosis [**69**], reducing the difficulty of the complex signal equation. Furthermore, in most cases, attributing a single T2* value for all lipid resonances is acceptable, but field inhomogeneity effects are to be expected. However, each lipid resonance can be given a specific T2* value [**70**] for better field homogeneity.

The proton density-weighted estimation of water and lipid reflects the metabolite concentration properly, when the correction of relaxation effects and of other system and field effects is executed correctly. 

Although independent from confounders such as glycogen, fibrosis, and hemochromatosis, MRS requires patient co-operation. Despite all these, it provides a good degree of precision when it comes to the quantitative analysis of the hepatic FF. 

With all these available MR imaging techniques, MR spectroscopy must be mentioned as an invaluable becoming tool in the assessment of NAFLD. 

## Conclusions

In conclusion, NAFLD is an ailment to be reckoned with, as its increase at a global scale is worrisome. Hepatic FF assessing by using non-invasive methods such as US, CT, MRI and MRS has given physicians great insight into NAFLD. The diversity of current imaging, non-invasive alternatives of quantifying the degree of hepatic steatosis is a breath of fresh air for both physicians and patients. Each alternative has advantages and disadvantages and choosing the appropriate one must be done keeping in mind the patient’s best interest. To sum up, there are pros and cons of the most frequently used imagistic methods to determine the degree of fat content in the hepatic parenchyma.

The necessity for precise, non-invasive, and reproducible imagistic methods for estimating the lipid content of the liver is extremely high as patients need to be evaluated periodically and efficiently assessed in order to determine the treatment results and the lifestyle changes used to manage hepatosteatosis. This high demand will surely result in great innovation in the field of non-invasive imagining, improving the current imagistic methods for evaluating NAFLD and hopefully will even completely replace the use of liver biopsy. 

**Acknowledgments**

This work was supported from the research grant entitled “Complex-imaging evaluation of liver steatosis in patient with combined Sylimarin, Phyllanthus niruri and choline treatment”, contract number 1334/ 17.12.2015. 
